# Theoretical Investigation of Early Cancer Biomarker Sensing Using a PMMA–Gold Hybrid Quasi-D-Shaped Photonic-Crystal-Fiber-Based Surface Plasmon Resonance Biosensor

**DOI:** 10.3390/mi17010068

**Published:** 2025-12-31

**Authors:** Ayushman Ramola, Amit Kumar Shakya, Nezah Balal, Arik Bergman

**Affiliations:** Department of Electrical and Electronics Engineering, Ariel University, Ariel 40700, Israel; ayushmanr@ariel.ac.il (A.R.); nezahb@ariel.ac.il (N.B.); arik.bergman@msmail.ariel.ac.il (A.B.)

**Keywords:** quasi D shaped, photonic crystal fiber (PCF), surface plasmon resonance (SPR), polymethyl methacrylate (PMMA), cancer

## Abstract

In this work, a quasi-D-shaped photonic crystal fiber (PCF)-based surface plasmon resonance (SPR) biosensor is proposed and numerically investigated using the finite element method (FEM) implemented in COMSOL Multiphysics version 6.2 for the detection of cancer cells with different refractive indices. The biosensor has a coating of plasmonic material gold (Au) and a polymer coat of polymethyl methacrylate (PMMA). The effects of plasmonic material thickness and air hole dimensions on key sensing parameters, including confinement loss (CL), wavelength sensitivity (WS), and amplitude sensitivity (AS), are systematically analyzed. The results revealed that increasing plasmonic thickness beyond its optimum value significantly raises CL while reducing sensitivity due to reduced penetration depth of the evanescent field. Similarly, variations in the geometrical dimensions of the air holes (±10%) also affect the sensor response, emphasizing the importance of precise structural optimization. For the optimized design the proposed biosensor exhibits high performance with a maximum WS of 31,000 nm/RIU for MDA-MB-231 cells under x-polarization and 29,500 nm/RIU under y-polarization. The corresponding resolutions achieved are as low as 3.22 × 10^−6^ RIU and 3.38 × 10^−6^ RIU, respectively, with AS exceeding 9000 RIU^−1^. The WS, AS, and other sensing parameters obtained from our sensor are relatively higher than some of the PCF–SPR sensors reported recently. These numerical results demonstrate that the optimized quasi-D-shaped PCF–SPR biosensor exhibits enhanced sensitivity to refractive index (RI) variations associated with cancerous cells, suggesting its suitability for early detection.

## 1. Introduction

**Figure 1:**
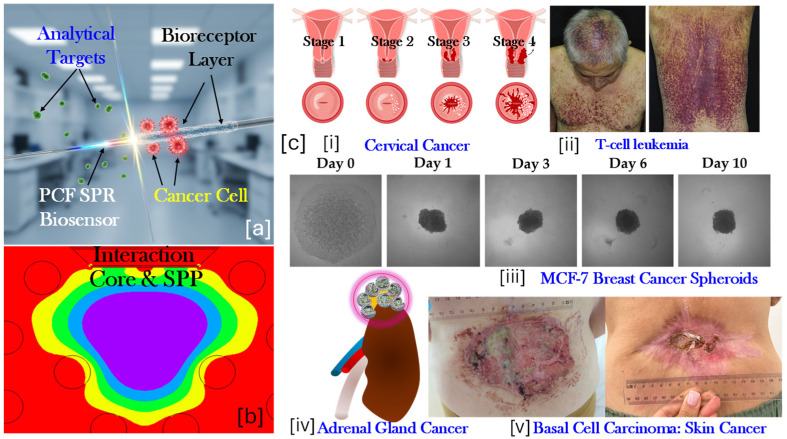
(**a**) Polymer-coated optical fiber with a bioreceptor layer designed to attract cancer cells and other analytical targets, (**b**) interaction between the cancer-cell-containing fluid, polymer layer, and plasmonic layer for cancer cell detection, and (**c**) cancer progression examples: (**i**) stages of cervical cancer progression [9], (**ii**) a body infected with T-cell leukemia [10], (**iii**) MCF-7 breast cancer spheroid progression from Day 1 to Day 10 [11], (**iv**) adrenal gland cancer, and (**v**) human skin affected by basal cell carcinoma [12].

Cancer is a complex disease developed due to the uncontrolled cell growth and has the potential to invade or spread to other parts of the body. It is considered as one of the leading causes of morbidity and mortality worldwide, accounting for nearly 10 million deaths in 2020, according to the World Health Organization (WHO) [1]. Cancers infect body parts like breast, lung, colorectal, prostate, skin, and stomach. Several forms of cancers such as cervical, ovarian, pancreatic, and hematological malignancies remain highly lethal due to late-stage diagnosis [2]. Despite tremendous progress in oncology, cancer-related medications, and cancer surgeries, the global burden of cancer continues to rise, largely driven by several parameters such as aging populations, genetic predispositions, environmental exposures, lifestyle factors, and infections (e.g., human papillomavirus (HPV) in cervical cancer, hepatitis B virus (HBV) and hepatitis C virus (HCV) in liver cancer, and epstein barr virus (EBV) in certain lymphomas [3]. The high mortality rate is often due to the difficulty of detecting cancer at an early stage. Many cancers remain unknown in the initial phases and are typically diagnosed only after progression to advanced or metastatic stages. For example, the survival rate of breast cancer is over 90% when detected early but drops drastically in the later stages [4]. Similarly, pancreatic and ovarian cancers exhibit poor prognosis primarily due to the absence of reliable early biomarkers and screening tools. Traditional diagnostic methods include biopsy, histopathology, immunohistochemistry, ELISA assays, and medical imaging (MRI, CT, PET), which have been considered the gold (Au) standard for cancer diagnosis [5]. However, these techniques are often invasive, time-consuming, expensive, and limited in sensitivity, particularly for detecting trace levels of circulating cancer biomarkers such as proteins, DNA fragments, exosomes, and circulating tumor cells (CTCs) [6]. This has driven growing interest in biosensor-based platforms that can detect cancer biomarkers directly in biological fluids such as blood, serum, urine, or saliva [7,8]. Figure 1a represents the prototype of an optical fiber which can be used to detect cancer biomarkers through the bioreceptor layer installed over it. Figure 1b represents the interaction of the core and the excited surface plasmon polaritons (SPPs) along the metal–dielectric interface. Figure 1c represents the growth of the cervical cancer from stage 1 to stage 4, a human body affected by T-cell leukemia, MCF 7 breast cancer progression from Day 1 to Day 10, a cancer cell infecting the adrenal gland, and basal cell carcinoma (BCC) causing skin cancer [9,10,11,12]. Thus, there is an urgent need for non-invasive, cost-effective, and highly sensitive diagnostic technologies capable of detecting cancer at its earliest.

Photonic crystal fiber (PCF)-based surface plasmon resonance (SPR) biosensors have emerged as one of the most promising classes of optical sensors efficient in the early cancer detection due to their ability to confine light in microstructured geometries while allowing strong interaction with the plasmonic layer and bio fluid channel [13].

In PCF–SPR sensor, the evanescent field of the guided mode couples with surface plasmons at the interface between a dielectric and a metallic layer, resulting in a sharp resonance condition that is highly sensitive to refractive index (RI) variations caused by biomolecular binding [14]. This principle enables the detection of cancer biomarkers, proteins, nucleic acids, and even whole cells in a label-free and real-time manner. One of the important components of PCF–SPR biosensors is the plasmonic materials. Several plasmonic materials like Au and silver (Ag) have been used in SPR sensor from early stages. Au is the most widely used plasmonic material due to its excellent chemical stability, biocompatibility, and ability to sustain strong surface plasmons in the visible to near-infrared spectrum. Ag exhibits sharper resonance and higher sensitivity but suffers from oxidation and poor stability in biological environments. Due to which there is rise in alternatives materials such as aluminum (Al) and copper (Cu), beside emerging 2D materials like graphene, graphene oxide, and MXenes that have also been investigated for enhancing sensitivity and extending detection ranges [15]. Nowadays hybrid designs that combine noble metals with polymers like PMMA, polyvinyl alcohol (PVA), polydimethylsiloxane (PDMS), polyethylene glycol (PEG), chitosan, SU-8, dielectric overlayers, or nanocomposites have gained attention for improving both stability and selectivity [16,17,18]. PCF–SPR biosensors models can be classified based on their structural configurations. Conventional circular PCFs utilize microstructured air holes surrounding a solid or hollow core, while quasi-D-shaped PCFs provide a flat polished region for easier metal and polymer deposition, leading to stronger light–plasmon coupling. Hollow-core PCFs allow infiltration of analytes directly into the fiber core, increasing interaction length, whereas solid-core PCFs rely on evanescent field penetration at the cladding–metal interface. Further variations include dual-core PCFs for differential sensing, grating-assisted PCFs for wavelength tuning, and hybrid PCF designs that incorporate functional polymers, dielectric layers, or nanomaterials to enhance performance. Internal metal deposition (IMD) [19], external metal deposition (EMD) [20], D-shaped PCF [21], the merger of EMD and D-shaped [22], and the merger of IMD and EMD are some prominent PCF–SPR sensor models [23]. Abdelghaffar et al. [24] presented a V-shaped PCF biosensor based on SPR with ZrN coating for cancer detection. Using finite element analysis, the optimized design achieves high sensitivities up to 6214 nm/RIU for breast, basal, and cervical cancer cells. Sardar et al. [25] introduces a dual-side polished PCF–SPR biosensor with Au via chemical vapor deposition (CVD) for cancer cell detection. They designed and optimized sensor using FEM. The sensor achieves high performance with 7143 nm/RIU sensitivity, 2.9 × 10^−5^ RIU resolution, and a figure of merit (FOM) of 900, offering a precise, low-cost tool for early cancer diagnostics.

The proposed biosensor introduces several innovative features that significantly advance plasmonic biosensing performance for cancer detection. The novelty of the proposed work lies in the integrated design and optimization that employs a hybrid plasmonic–polymer coating for highly sensitive detection of cancer cells. Unlike previous SPR studies that primarily used either plasmonic or dielectric layers alone, in the proposed research a dual layer of Au and PMMA along with Zeonex is used, which enhances the plasmonic resonance by several factors, improving field confinement, biocompatibility, and stability of the sensing region. The sensor achieves an ultra-high wavelength sensitivity (WS) of up to 31,000 nm/RIU, enabling highly precise discrimination of cancer cell RI variations. In addition, an extremely low detection limit allows the identification of minute changes in cancer cell fluids, which is crucial for early-stage diagnostics. Furthermore, a detailed parametric analysis of plasmonic layer thickness and air-hole dimensional variations (±10%) has been carried out to understand their effects on the sensor parameters leading to an optimized configuration that achieves an extremely high sensing parameter. Unlike conventional PCF–SPR sensors that operate effectively under a single pol., the proposed design demonstrates high AS, exceeding 9 × 10^3^ RIU^−1^ under both x- and y-pol. modes, ensuring independent pol. and robust operation. Collectively, these features establish the proposed biosensor as a reliable, tunable, and high-performance platform for advanced cancer diagnostics applications. Compared to some recently reported sensor, our sensor model exhibits superior performance. For instance, ref. [26] reported a WS of 10,714 nm/RIU and SR in the order of 10^−6^ RIU, for x-pol., while ref. [27] achieved a WS of 10,000 nm/RIU for y-pol. Some experimental articles also reported a high WS of 28,100 nm/RIU based on Au coated D-shaped fiber [28], a WS of 29,000 nm/RIU based on the dual coat of Au TiO_2_ [29], etc. In contrast, our design reported even higher sensing parameter values for individual cancer cell compared to recent sensor models.

Overall, this work presents a novel and efficient PCF–SPR biosensor architecture that combines hybrid material design, structural optimization, and biomedical applicability, offering an improved performance and practically potential for early cancer detection.

## 2. Experimental Setup for Obtaining Refractive Index of Various Cancer Cells

**Figure 2:**
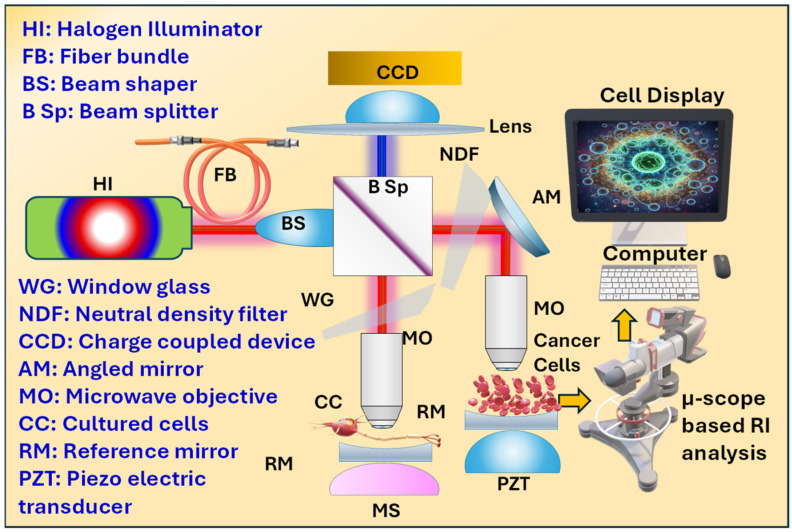
Experimental setup to obtain RI values of various cancerous cells.

The RI of the various cancer cells can be obtained from the experimental setup as presented in Figure 2, which is a form of a digital holographic microscopy (DHM) system that is used to determine the RI of various cancer cells by using light interference [30]. A halogen illuminator (HI) generates light, which is split by a beam splitter (B Sp) into an object beam and a reference beam. The object beam passes through the cultured cells (CC), and the cancer cells’ dense internal material causes a unique phase shift in the light proportional to their RI. The reference beam reflects off a mirror. The B Sp then recombines the two beams, creating an interference pattern (a hologram) that is captured by a charge-coupled device (CCD) camera. A computer analyzes this hologram using specialized algorithms to calculate the optical path difference (OPD) and, subsequently, the RI of the cells, allowing for the differentiation of cancer cells, which typically exhibit a higher RI.

### Working and Component Description of the Proposed Setup

In this optical setup, light from the HI is first guided through a fiber bundle and a beam shaper, which together produce a spatially uniform beam suitable for interferometric imaging. This well-collimated light enters the interferometer and is divided by a beam splitter into two distinct paths: the sample arm and the reference arm. In the sample arm, light passes through a microscope objective that focuses it onto the biological specimen, such as cancer cells cultured on a coverslip, while in the reference arm, an identical objective focuses light onto a highly reflective reference mirror. The reflected light from both the sample and reference mirror retraces their paths and recombines at the beam splitter, producing an interference pattern whose intensity depends on the relative optical phase difference between the two arms. To extract quantitative information, the reference mirror is mounted on a piezoelectric transducer (PZT) that moves in precisely controlled steps, commonly 0, π/2, π, and 3π/2 radians, introducing known phase shifts between successive interferograms. This process, known as phase-shift interferometry, allows the system to record a sequence of intensity images corresponding to different phase offsets. Using computational phase reconstruction algorithms such as 3-step or 4-step phase-shifting methods, or Fourier-transform techniques, these intensity frames are mathematically combined to yield a phase map φ (x, y) that represents the spatial variation of the optical path difference (OPD) across the field of view. Finally, this phase map is converted into an optical thickness distribution, and with knowledge of the physical thickness of the cell layer t (x,y), the local RI n (x, y) of the cancer cells can be determined. The resulting RI map provides a direct, label-free measure of the cells’ internal composition and density, enabling differentiation among various cancer cell types based on their optical properties.

A detail explanation of the various components of the proposed setup along with their working is expressed as follows.

- Halogen Illuminator (HI): A broadband white light source having low spatial coherence, i.e., less speckle. Its disadvantages include a short temporal coherence, which requires small path-length mismatch to see fringes, unless a bandpass filter is used.
- Fiber Bundle (FB): It works to moves the lamp output to the instrument input and homogenizes light. Fiber output gives more uniform illumination.
- Beam Shaper (BS): It produces the desired illumination profile for the sample being investigated (e.g., flat-top or controlled gaussian) so the field at the objective pupil is uniform.
- Beam Splitter (B Sp): It splits light into two different paths, i.e., the sample arm and reference arm, and later recombines the reflected beams. In a Linnik interferometer the same splitter handles both directions.
- Window Glass (WG): It is also widely known as a cover slip, in which the sample is typically grown on a coverslip (glass window) that the objective focuses through. The coverslip and its index must be accounted for in path length calculations and calibration.
- Neutral Density Filter (NDF): It works to attenuate illumination to protect the detector and avoid camera saturation. It plays a very important role in protecting the sample from excessive light.
- Angled Mirror (AM): It is a kind of folding mirror that is used to route light and to align the beam toward the microscope arms or camera.
- Microscope Objectives (MOs): The schematic that shows identical objectives in both arms that is the hallmark of a Linnik interferometer where identical high-NA objectives minimize aberration differences between arms. These focus the beam onto the sample cells in one arm and onto the reference mirror in the other arm.
- Reference Mirror (RM): It is a flat mirror in the reference arm mounted on a PZT for precise axial motion (phase shifting). The PZT moves the mirror by known fractions of a wavelength to implement phase-shift interferometry.
- Piezoelectric Transducer (PZT): It produces the calibrated mirror displacements (e.g., λ/4 steps) used for phase-shifting algorithms.
- Cultured Cells (CCs): The cells on the coverslip in culture medium produce a local optical path difference versus the surrounding medium.
- Microscope Stage (MS): Its function is to hold the sample; often motorized for scanning the cultured cells.
- Lens + CCD: The recombined interference pattern is imaged onto the CCD by a tube lens or relay. The CCD records interferograms or the intensity images of the cancer cell samples which are later used for phase retrieval.

When light passes through or reflects from a cancer cell, it experiences a delay relative to the same light travelling through the surrounding medium (culture fluid). This delay appears as a phase shift (φ) in the interference pattern recorded by the CCD. Mathematically it is expressed by Equation (1) [31].

(1) $$\emptyset \left(x,y\right)=\frac{4\pi }{\lambda }\left(n_{cell}\left(x,y\right)-n_{medium}\right)t(x,y)$$

where λ is the wavelength of the light source, $n_{medium}$ is the RI of the cultural medium, $n_{cell}\left(x,y\right)$ is the RI of the cancer cells, t(x,y) represents the local thickness of the cells, and $\emptyset \left(x,y\right)$ represents the measured phase map of the cells. Therefore, once we have calculated the phase map $\emptyset \left(x,y\right)$ and cell thickness t(x,y), the optical phase delay can be measured into a local RI of the cell and expressed by Equation (2) [31].

(2) $$n_{Cell}\left(x,y\right)=n_{medium}+\frac{\lambda \emptyset (x,y)}{4\pi t(x,y)}$$

Cancer progression changes cell composition, water content, and macromolecular density. These microscopic changes alter $n_{Cell}$ and this change is caused further due biological reasons, as described in Table 1.

Thus, the RI values of the various cancer cell types can be experimentally obtained, which can be further used to design a plasmonic SPR sensor for several medicinal applications.

## 3. Design and Operating Principle of the Proposed Biosensor

The key design parameters and materials selected for the proposed biosensor is supported by both physical reasoning and numerical validation. Au is selected as the plasmonic material owing to its strong and stable SPR behavior in the visible–NIR region, while PMMA is incorporated as an intermediate polymer layer to enhance field confinement and biocompatibility. The quasi-D-shaped geometry is selected to provide efficient cancer cell fluid access and stronger light–matter interaction compared to conventional circular PCFs. Thus, various details of the proposed biosensor are presented in following subsections.

### 3.1. Geometrical Modelling of the Proposed Biosensor Design

**Figure 3:**
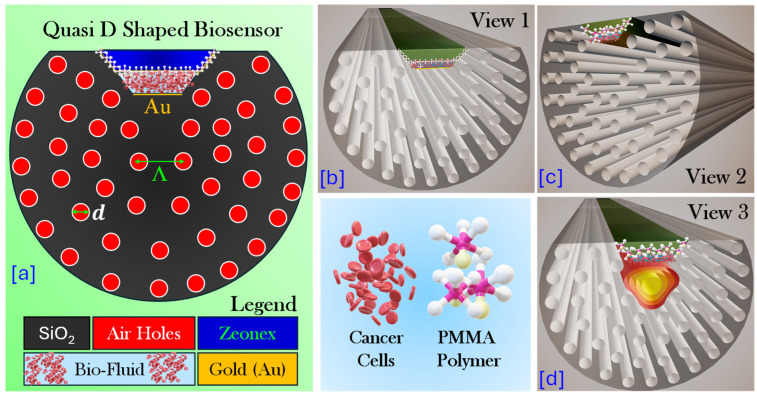
(**a**) Schematic representation of the proposed quasi-D-shaped PCF–SPR biosensor for cancer screening, with a cross-sectional view highlighting the PMMA polymer layer, air holes (red), Au-coated D-shaped sensing region, with pitch (Λ) and air hole diameter (*d*); (**b**) 3D front view of the proposed PCF; (**c**) 3D side view of the proposed PCF; and (**d**) 3D front view of the proposed PCF illustrating the light propagation into the fiber and its interaction within the sensing region, leading to SPR excitation.

Figure 3a represents the geometrical model of the proposed quasi-D-shaped PCF–SPR biosensor. The base structure of the PCF is formed in a silica (SiO_2_) background, shown in black. The fiber contains periodic arrangement of air holes represented by red circles that are arranged in a hexagonal lattice. The air hole diameter marked as d is equal to 1 μm. A parametric study is performed by varying the Au thickness from 40 nm to 60 nm. The optimum thickness of 45 nm is identified based on the maximum WS and amplitude sensitivity (AS) achieved at this value. For comparison, the sensor performance at a thickness of 55 nm is also analyzed and presented in a later section, illustrating how the sensing parameters are affected when the plasmonic layer thickness deviates from the optimum value.

The pitch (Λ) represents the center-to-center distance between adjacent air holes and is equivalent to Λ = 2.5 μm. These air holes confine and guide light within the fiber core by modifying the effective RI. The top portion of the PCF is polished into a quasi-D shape, exposing a flat surface where the plasmonic and polymer layers are deposited. On this polished region a thin Au layer of 45 nm film is deposited as the plasmonic material. On top of the Au layer a trapezoidal biofluidic sensing region is presented. The length of the trapezoidal upper side is 3.98 μm while the length of the lower side is 2.98 μm. The distance between the upper side and lower side of the trapezoid is 0.5 μm. A thin layer of polymer PMMA is installed over the sensing layer having the geometrical thickness of 60 nm. Finally, over the PMMA layer a trapezoidal layer of the polymer Zeonex is installed to make the PMMA layer intact. The length of the trapezoidal upper side is 5.9 μm while the length of the lower side is 4.0 μm. The distance between the upper side and lower side of the trapezoid is 0.945 μm. The polymer coatings (PMMA or Zeonex) are applied, to serve both as a functional layer and for bio-receptor immobilization. The bio-fluid (cancer cell fluid) directly interacts with this layered structure, enabling efficient SPR coupling. Figure 3b–d represents three different views of the three-dimensional (3D) geometry of the proposed sensor model.

### 3.2. Materials Description Used in the Proposed Biosensor

The proposed biosensor model is crafted with the assistance of background material SiO_2_, plasmonic material Au, freely available air, and the polymers PMMA and Zeonex [38,39]. First the RI of the background material SiO_2_ is expressed using the Sellmeier equation represented by Equation (3) [40].

(3) $$n^{2}\left(\lambda \right)=1+\frac{B_{1}\lambda^{2}}{\lambda^{2}-C_{1}}+\frac{B_{2}\lambda^{2}}{\lambda^{2}-C_{2}}+\frac{B_{3}\lambda^{2}}{\lambda^{2}-C_{3}}$$

where n(λ) is defined as the RI at wavelength λ, while $B_{1}$, $B_{2}$, $B_{3}$, $C_{1}$, $C_{2}$, and $C_{3}$ are the Sellmeier coefficients. The value of these constants for SiO_2_ are expressed as $B_{1}=0.69616300$, $B_{2}=\;$0.407942600, $B_{3}=\;$0.897479400, $C_{1}=$ 4.67914823 $\times \;10^{-3}\;{\mu m}^{2}$, $C_{2}=1.35120631\times 10^{-2}\;{\mu m}^{2}$, and $C_{3}=\;97.9340025\;{\mu m}^{2}$.

The relative permittivity of the Au is expressed using the Drude Lorentz model presented by Equation (4) [40].

(4) $$\varepsilon_{Au}=\epsilon_{\infty }-\frac{\omega_{p}^{2}}{\omega \left(\omega +i\gamma_{D}\right)}+\sum_{j}\frac{S_{j}\omega_{0j}^{2}}{\omega_{0j}^{2}-\omega^{2}+i\omega \gamma_{j}}$$

where $\varepsilon_{Au}$ represents the permittivity of Au, $\epsilon_{\infty }$ is the permittivity at high frequency, ω represents the angular frequency and expressed as ω=2πc/λ, $\omega_{p}^{2}$ represents plasma frequency, $\gamma_{D}$ is the damping frequency, and $S_{j}$, $\omega_{0j}^{2}$, and $\gamma_{j}$ represent damping constants for $j^{\mathrm{th}}$ Lorentz oscillator. The other parameters of the expression are $\frac{\gamma_{D}}{2\pi }=15.92\;THz$, $\frac{\omega_{P}}{2\pi }=2113.6\;THz$, and $\omega_{0j}=650.07\;THz$, and finally, the spectral width is denoted as $\frac{\omega \gamma_{j}}{2\pi }=104.86\;THz$.

The RI of air is 1.000293 RIU, as obtained from the Ciddor equation [41].

The RI of the polymer PMMA is also determined by the Sellmeier equation as expressed by Equation (5) [42].

(5) $$n_{PMMA}=C_{1}+C_{2}\lambda^{2}+C_{3}\lambda^{-2}+C_{4}\lambda^{-4}+C_{5}\lambda^{-6}+C_{6}\lambda^{-8}$$

where $C_{1}=2.399964$, $C_{2}=$ −8.308636 × $10^{-2}$, $C_{3}=$ −1.919569 × $10^{-1}$, $C_{4}=$ −8.720608 × $10^{-2}$, $C_{5}=$ −1.666411 × $10^{-2}$, and $C_{6}=$ 1.169519 × $10^{-3}$.

PMMA offers several advantages when employed in PCF–SPR sensors. It exhibits excellent optical transparency in the visible and near-infrared regions, ensuring efficient light guidance and strong plasmonic excitation. Its RI (~1.49) enables effective phase matching between the core-guided mode and surface plasmon mode, which is essential for high sensitivity. PMMA is cost-effective, easy to process, and compatible with various microfabrication techniques, making it suitable for fabricating complex PCF structures. Moreover, it is biocompatible, chemically stable, and lightweight, which are desirable properties for biosensing applications. The surface of PMMA can also be functionalized or doped with dyes, nanoparticles, or polymers to enhance selectivity and sensitivity, further improving the performance of PCF–SPR sensors [43,44].

Finally, a trapezoidal shaped layer of Zeonex is installed over the biofluidic channel, which is to sandwich the PMMA and biofluidic layer with the SiO_2_ material. Zeonex is scientifically known as cyclo-olefin polymer (COP) [45]. Zeonex offers several distinct advantages over other polymers: (i) it possesses a low material absorption loss of 0.2 cm^−1^, (ii) it has a stable RI of 1.53, (iii) it has excellent suitability when used for biosensing applications, (iv) it possess a high glass transition temperature, and (v) its insensitivity to humidity [46].

### 3.3. Mesh Description of the Proposed Biosensor

In the proposed biosensor six different design components are present, which are meshed with different meshing conditions to obtain accurate, stable, and efficient solutions. The biosensor meshing computation is performed through the FEM-based COMSOL software version 6.2 package [47]. Table 2 presents the details about the element sizes used to mesh each design component of the proposed biosensor.

**Figure 4:**
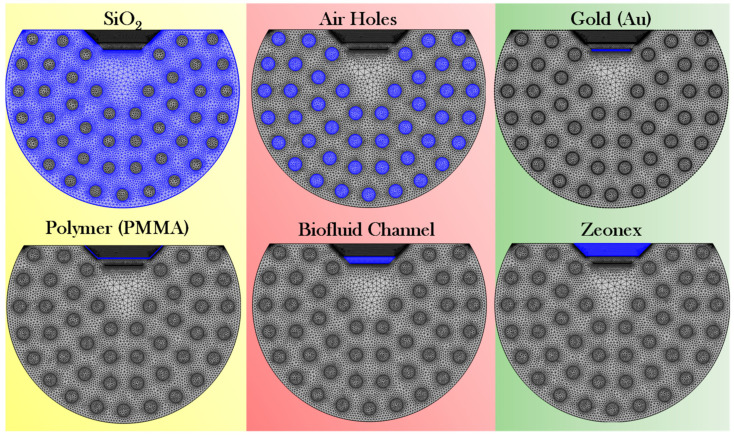
Mesh configuration of the different sensor domains of the proposed biosensor.

Figure 4 presents the pictorial view of the meshing condition of the different biosensor components. In the meshing condition, the different components are meshed using user-controlled free triangular meshing conditions. SiO_2_ and air holes are meshed using normal mesh. Au, PMMA, the biofluidic channel, and the Zeonex layer, are meshed through extremely fine meshing conditions. The complete mesh consists of 13,501 mesh vertices, 26,565 triangles, 2018 edge elements, and 201 vertex elements. The domain element statistics contain following details: number of elements = 26,565, minimum element quality = 0.5247, average element quality = 0.8661, element area ratio = 3.56 × 10^−7^, and mesh area = 207 μm^2^. Different mesh configurations are used to mesh design elements to achieve an optimal balance between computational efficiency and simulation accuracy. Finally, the sensor structure is simulated applying the scattering boundary condition (SBC).

### 3.4. The Role of Polymer Materials in the Surface Plasmon Resonance Sensing Application

The polymers play a vital role in the PCF-SPR sensing applications. There are different polymers that can be used in the sensor model for sensing applications. Some of the notable polymers include PMMA [48], Cyclo-olefin copolymer (COC) [49], polyethylene (PE/HDPE) [50], polytetrafluoroethylene (PTFE/Teflon) [51], MR-PMMA (methyl-red doped PMMA) [52], and SU-8 (epoxy-based polymers) [53]. Polymers possess the following important features, including the fat polymers play a multifaceted role in PCF-based SPR sensors, serving as both the structural and active components of the fiber. Polymers are commonly employed as background or cladding materials due to their low optical loss, suitable RI for phase matching, and negligible material dispersion, which together help maintain stable SPR. Polymers can also act as active sensing layers when doped with dyes or photochromic molecules, responding to external stimuli like light, chemicals, or biomolecules by altering their optical properties [52]. Thin polymer coatings on the metal layer enhance the evanescent field overlap with the analyte, allowing precise tuning of the SPR resonance wavelength (RW) and improving sensitivity. Additionally, polymers provide mechanical flexibility and chemical resilience, facilitating the fabrication of microstructured fibers and the incorporation of functional materials without compromising the fiber structure. Most of the polymers are biocompatible and chemically inert, making them suitable for biosensing applications, and their surfaces can be functionalized to increase selectivity toward specific targets. Finally, polymers exhibit good thermal and environmental stability, ensuring reliable sensor performance under typical operating conditions.

### 3.5. Surface Plasmon Resonance Principle of the Proposed Biosensor

SPR is a phenomenon that arises at the interface between a metal and a dielectric when incident light excites collective oscillations of free electrons, producing SPPs [54]. In PCF–SPR biosensor, light propagates through the fiber core, generating an evanescent field that penetrates the thin metallic layer deposited on the fiber surface or within microstructured air holes. The excitation of SPPs occurs under the phase-matching condition, where the propagation constant of the core-guided mode ($\beta_{core}$) matches that of the SPP mode ($\beta_{SPP}$) at the metal dielectric interface and is expressed by Equation (6) [55].

(6) $$\beta_{core}=\beta_{SPP}=\frac{2\pi }{\lambda }\sqrt{\frac{\varepsilon_{m}\varepsilon_{\alpha }}{\varepsilon_{m}+\varepsilon_{\alpha }}}$$

where λ is the wavelength of light, $\varepsilon_{m}$ is the complex dielectric constant of the metal, and $\varepsilon_{\alpha }$ is the dielectric constant of the biofluid. Under this phase-matching condition, energy from the guided mode is efficiently transferred to the surface plasmon, producing a characteristic resonance dip in the transmitted spectrum. Any change in the biofluid RI ($∆n_{a}$) modifies $\varepsilon_{\alpha }$, thereby shifting the RW according to Equation (7) [56].

(7) $$RW\propto ∆n_{a}$$

When light propagates through the core of a PCF, it is primarily guided by total internal reflection (TIR) due to the higher RI of the core compared to the surrounding cladding or air holes. At the core-cladding interface, the electromagnetic field does not abruptly vanish; instead, a portion of the field extends beyond the core as an evanescent wave, decaying exponentially into the lower-index region. Mathematically, the evanescent field in a dielectric region can be expressed by Equation (8) [56].

(8) $$E\left(z\right)=E_{0}e^{-kz}$$

where $E_{0}$ is the electric field amplitude at the interface, *z* is the distance from the core boundary, and k is the evanescent decay constant, which depends on the RI, as presented by Equation (9) [57].

(9) $$k=\frac{2\pi }{\lambda }\sqrt{n_{core}^{2}-n_{clad}^{2}}$$

Here, $n_{core}$ and $n_{clad}$ are the RI of the fiber core and surrounding medium, respectively, and λ represents the wavelength of light.

Finally, Zeonex serves as the fiber background, providing a stable RI contrast and low optical loss, while thin PMMA polymer coatings can enhance evanescent field overlap and improve sensitivity. The geometry of the PCF, including core size, air-hole arrangement, and metal layer thickness, plays a crucial role in controlling the evanescent field distribution, optimizing phase matching, and maximizing the SPR response. This configuration allows compact, highly sensitive, and tunable biosensing suitable for detecting small changes in RI caused by chemical or biological interactions. Table 3 presents details about the various cancer cells along with their RI values.

## 4. Methodology and Analysis of Findings

### 4.1. Core Configuration and SPP Mode Profile of the Proposed Biosensor

**Figure 5:**
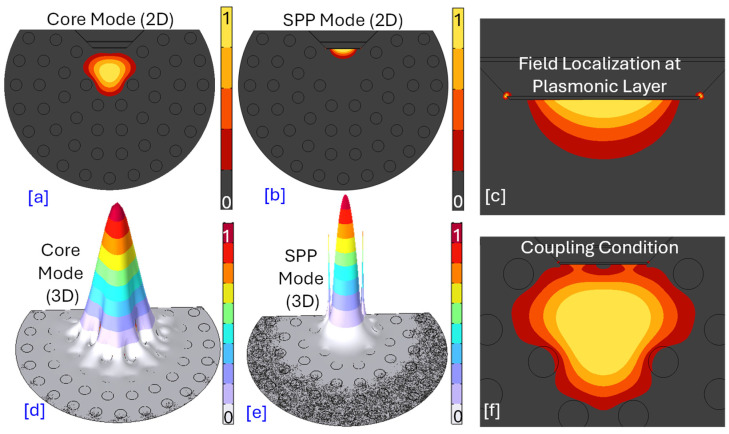
Field distribution intensity profile of the proposed sensor for MCF 7 cancer cells with an RI = 1.401 (**a**) Cross-sectional images showing the core mode intensity profiles in 2D for different light confinement mechanisms. (**b**) Cross-sectional images showing the SPP mode intensity profiles in 2D for different light confinement mechanisms. (**c**) SPP mode field localization intensity profile. (**d**) Core mode intensity profile in 3D. (**e**) SPP mode intensity profile in 3D. (**f**) Coupling condition of the core mode and SPP mode for the proposed biosensor.

The core mode, SPP mode, and plasmonic behavior of the sensor model are represented in Figure 5.

Figure 5a,d represents the 2D and 3D profiles of the proposed biosensor that exhibits a highly localized electromagnetic field profile. The color gradient clearly shows the strongest field concentrated right in the center of the structure, tapering off rapidly as it moves outwards. The 3D view further emphasizes this central peak, resembling a well-contained wave packet. In a PCF-SPR sensor, the core mode represents the light guided within the fiber’s core. This is the propagating optical mode that carries the input signal. Its strong confinement ensures efficient transmission of light through the fiber. Figure 5b,e represents the 2D and 3D profiles of the SPP mode of the proposed biosensor. While there might be some central presence, the defining characteristic is the field’s strong localization at the interface where the plasmonic material Au interact with the cancerous biofluid. The SPP mode is an electromagnetic wave that propagates along the interface between a metal and a dielectric (often the sensing medium). In a PCF–SPR sensor, a thin layer of plasmonic metal is typically deposited on the inner surface of the air holes or the outer surface of the fiber, but in D-shaped fiber plasmonic material it is deposited on the polished surface. The SPP mode is excited at this metal–dielectric interface. Its field is highly sensitive to changes in the RI of the surrounding dielectric medium. Figure 5c represents the “field localization at plasmonic layer,” which perfectly aligns with the nature of an SPP. The field strength is highest right at this interface and decays evanescently away from it. Figure 5f highlights the “coupling condition,” which is critical for the operation of an SPR sensor. The core of PCF–SPR sensing lies in the coupling between the fiber’s core mode and the SPP mode. This coupling occurs when the phase matching condition is met, meaning the effective RI (or propagation constant) of the core mode becomes equal to that of the SPP mode. At this point, energy is efficiently transferred from the core mode to the SPP mode, leading to a dip or peak in the transmitted or reflected spectrum. This resonance condition is extremely sensitive to changes in the RI of the medium surrounding the plasmonic layer, making it an excellent mechanism for sensing.

Video S1 is provided as a Supplementary Materials to illustrate the electromagnetic field distributions of modes corresponding to x- and y-pol. The movie dynamically visualizes both low- and higher-order modes, highlighting their propagation behavior, field confinement, and resonance dynamics within the PCF–SPR sensor structure, while enabling comparative analysis under varying external RI conditions.

### 4.2. Analysis of the Biosensor Parameters

#### 4.2.1. Calculation of the Confinement Loss

Confinement loss (CL) in a PCF–SPR sensor quantifies how much optical power is lost from the guided mode in the core as light propagates. It primarily arises due to (a) the leakage of the core mode into the cladding or surrounding medium and (b) coupling to lossy SPP at the metal–dielectric interface. It is measured in dB/cm concerning PCF–SPR sensors. Physically CL is dependent on the RI of the surrounding medium (cancerous biofluid), geometry of the PCF (core size, air holes, metal layer thickness), and wavelength of incident light. It can be expressed by Equation (10) [66].

(10) $$CL=8.686\times \left(2\pi /\lambda \right)\times Im\left(n_{eff}\right)\times 10^{4}\;[dB/cm]$$

where λ represents wavelength of the incident light and $Im(n_{eff})$ is the imaginary part of the effective RI of the core mode.

The coupling of the core mode and SPP mode takes place at the RW reaching its maximum value, as summarized in Table 4. Distinction between various cancer biomarkers can be achieved by analyzing their characteristic core mode wavelengths under x-pol. and y-pol., respectively.

The results in Table 4 show that each cancer cell type, characterized by its specific RI, produces distinct resonance behaviors for the proposed biosensor. As the RI increases from 1.38 to 1.401, both the RW and CL shift toward higher values, indicating stronger light–plasmon coupling at higher RI. For each cancerous cell, the biosensor exhibits pol. dependent responses, with separate values of CL and RW for x- and y-pol. The differences between these pol., represented by |ΔCL| and |ΔRW|, provide information about the light behavior along different pol. For instance, Basal cells show a small RW shift of 3.0 nm between pol., while MCF-7 cells exhibit a much larger difference of 20 nm, reflecting the higher sensitivity of the sensor to variations in RI. These combined variations in RW and CL across different cancer cells act as unique optical fingerprints, enabling reliable distinction between blood components and specific cancerous biofluids.

**Figure 6:**
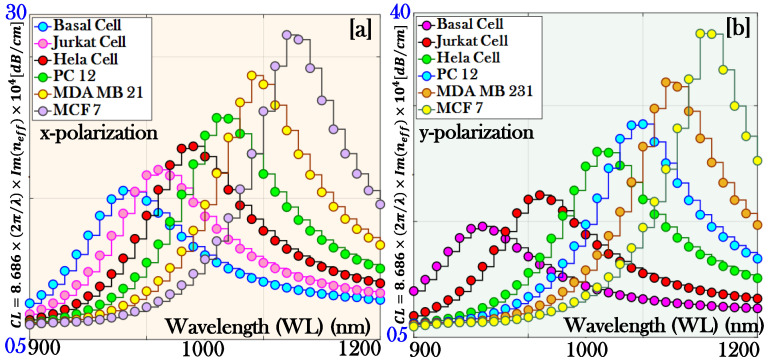
Confinement loss of the proposed biosensor: (**a**) x-polarization and (**b**) y-polarization.

Figure 6a,b illustrates the variation of CL for both x- and y-pol., clearly showing that the resonance behavior occurs at distinct RWs for each pol. state. This indicates that the coupling between the guided core mode and the surface plasmon mode is pol. dependent, leading to unique spectral signatures for different cancerous fluids. Such pol. specific responses are valuable, as they enhance the sensor’s ability to discriminate between various cancer cells.

#### 4.2.2. Calculation of the Wavelength Sensitivity

In PCF–SPR sensors, WS measures how much the RW shifts when the RI of the cancerous biofluid gets changed. It indicates how effectively the sensor can detect small RI changes, which is critical for biosensing applications. The WS of the biosensor is calculated using wavelength interrogation technique. From Table 4, details about the change in the RW can be obtained for various cancerous cells. The change in the WS can be obtained from Equation (11) [66].

(11) WS =∆RW/∆RI [nm/RIU]

where ∆RW indicates the change developed in the RW of two consecutive cancer cells and ∆RI represents the change in their consecutive RI values.

For the x-pol. mode, the RW of the basal cell is 954 nm, while that of the Jurkat cell is 975 nm. The wavelength shifts between the two is therefore 975 − 954 = 21 nm. Given a RI difference of 0.01, the corresponding WS is calculated as WS = 21/0.01 = 2100 nm/RIU.

Thus, the WS of 2100, 14,500, 13,666.66, 13,750, and 31,000 nm/RIU is obtained for the Basal, Jurkat, Hela, PC 12, and MDA MB 231 cells, respectively, corresponding to x-pol. Similarly, the WS of 1800, 12,500, 12,666.66, 12,750, and 29,500 nm/RIU is obtained for the Basal, Jurkat, Hela, PC 12, and MDA MB 231 cells, respectively, corresponding to y-pol.

Thus, the average WS of 15,003.33 nm/RIU and 13,843.33 nm/RIU is obtained corresponding to x-pol. and y-pol., respectively.

#### 4.2.3. Calculation of the Amplitude Sensitivity

**Figure 7:**
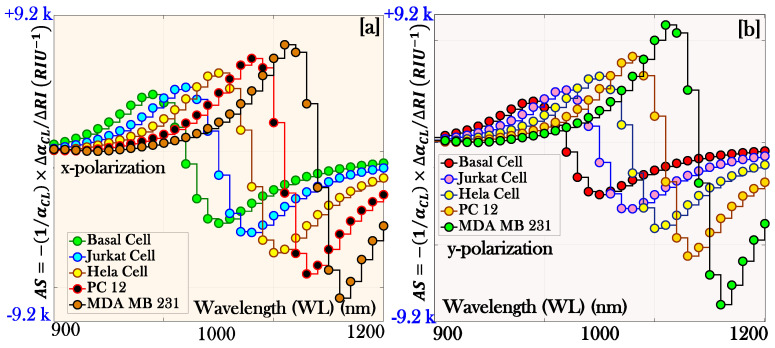
Amplitude sensitivity of the proposed biosensor: (**a**) x-polarization and (**b**) y-polarization.

Amplitude sensitivity (AS) refers to how strongly the transmitted (or reflected) light intensity changes in response to a small variation in the analyte’s RI. It is calculated using amplitude interrogation technique. It can be computed with the assistance of Equation (12) [66].

(12) $$AS=-\left(1/CL\right)\times ∆CL/∆RI\;[{RIU}^{-1}]$$

where ∆CL is the change developed in the *CL* of the proposed sensor. Figure 7a,b represents the behavior of the amplitude of the proposed biosensor for the x-pol. and y-pol., respectively.

The AS of 5057.97, 6193.26, 7490.46, 8202.36, and 9158.29 ${RIU}^{-1}$ is obtained corresponding to cancerous cells Basal, Jurkat, Hela, PC 12, and MDA MB 231, respectively, for x-pol. Similarly, the AS of 5017.46, 6083.76, 6990.49, 7862.54, and 9004.54 ${RIU}^{-1}$ is obtained corresponding to cancerous cells Basal, Jurkat, Hela, PC 12, and MDA MB 231, respectively, for y-pol. The peak value of the AS is obtained for the cancerous cell MDA MB 231 for both pol. modes.

#### 4.2.4. Calculation of the Sensor Resolution

Sensor resolution (SR) in a PCF–SPR biosensor is defined as the smallest measurable change in the RI of the biofluid that the sensor can detect accurately. It quantifies the detection limit of the sensor and depends on the sensor’s sensitivity and the precision of the measurement biosensing system. A lower value of resolution means the sensor can distinguish very fine variations in the biofluid RI. It can be expressed by Equation (13) [66].

(13) $$SR=∆RI\times {∆\lambda }_{min}/∆\lambda_{p\;}[RIU]$$

where ${∆\lambda }_{min}$ is defined as the minimum spectral resolution and is equivalent to 0.1 nm.

The calculated SR values for cancerous cells Basal, Jurkat, Hela, PC 12, and MDA MB 231 for x-pol. are 4.7619 × 10^−5^, 6.8965 × 10^−6^, 7.3170 × 10^−6^, 7.2727 × 10^−6^, and 3.2258 × 10^−6^, respectively. Similarly, the calculated SR values for cancerous cells Basal, Jurkat, Hela, PC 12, and MDA MB 231 for y-pol. are 5.5555 × 10^−5^, 8.0 × 10^−6^, 7.8947 × 10^−6^, 7.8431 × 10^−6^, and 3.3898 × 10^−6^, respectively. Thus, it can be observed that the proposed biosensor has obtained SR of the order 10^−6^, simply signifying the ability to detect a minimum detectable change in the order of 10^−6^.

#### 4.2.5. The Relationship Between Resonance Wavelength and Refractive Index of Cancer Cells

The relationship between RW and RI is the foundation of PCF–SPR sensing, as it directly enables RI measurement, defines sensitivity, and determines the overall performance of the biosensor. The relationship between RI and the shift in RW is crucial for the biosensor optimization. Parameters like root mean square error (RMSE), coefficient of determination (R^2^), and degrees of freedom for error (DFE), are usually used to interpret how well the relationship between the biosensor parameters is established [67].

**Figure 8:**
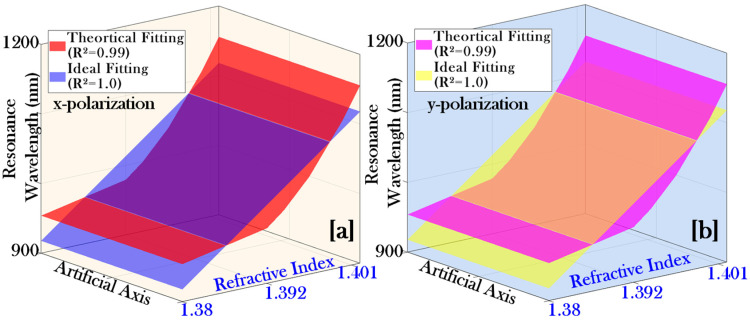
3D polynomial fitting of the sensor parameters resonance wavelength with refractive index: (**a**) x-polarization and (**b**) y-polarization.

Figure 8a,b represents the relationship between the biosensor parameters RI and RW corresponding to x-pol. and y-pol., respectively. Concerning Figure 8a,b, it represents the polynomial fitting expression as represented by Equation (14).

(14) $$f\left(RI\right)=p_{1}{\left(RI\right)}^{2}+p_{2}\left(RI\right)+p_{3}$$

The values of the polynomial constants $p_{1}$*,* $p_{2}$, and $p_{3}$ for x-pol. are presented in Table 5. The goodness of the fitting is expressed by the parameters SSE = 243.9841, R-square = 0.9922, DFE = 3.0, RMSE = 9.0182, and adjusted R-square = 0.9871.

Similarly, the values of the polynomial constants $p_{1}$*,* $p_{2}$, and $p_{3}$ for y-pol. are presented in Table 6. The goodness of the fitting is expressed by the parameters SSE = 216.5530, R-square = 0.9919, DFE = 3.0, RMSE = 8.4961, and adjusted R-square = 0.9864.

Therefore, for both x-pol. and y-pol., the coefficient of determination R^2^ values are found to be very close to 1.0. This indicates that the fitted model exhibits a good agreement with the simulated sensor data. Such high R^2^ values highlight the robustness and reliability of the fitting approach and confirm that the proposed PCF–SPR sensor structure maintains a strong correlation between the resonance response and the RI changes of the biofluids for both pol. states.

## 5. Analysis of Sensing Behavior Beyond the Optimum Thickness of the Plasmonic Material

### 5.1. Computation of the Sensor Sensing Parameters

**Figure 9:**
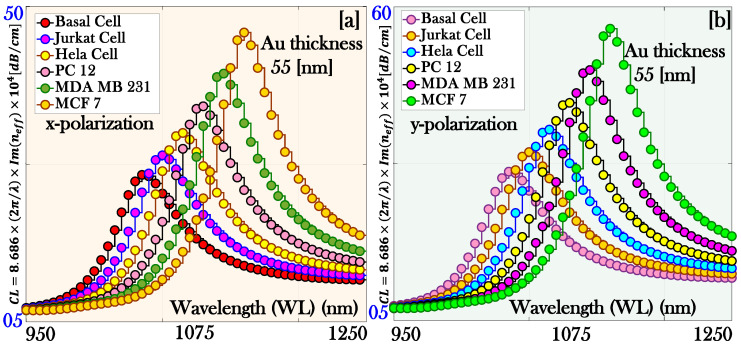
Confinement loss of the proposed biosensor with increased thickness of the plasmonic material Au (55 nm): (**a**) x-polarization and (**b**) y-polarization.

In this section, we have presented the result of the sensor parameters when we increase the thickness of the plasmonic materials beyond the optimum thickness. The optimum Au thickness is found to be 45 nm, while a thickness of 55 nm is also analyzed for comparative evaluation of sensor performance. The change in the sensor parameters CL is presented in Figure 9a,b, corresponding to x-pol. and y-pol., respectively. Table 7 quantifies the following changes in the CL for both pol. modes.

Thus, in a PCF–SPR sensor, CL plays a crucial role in determining sensor performance. However, if the CL becomes too high due to excessively thick plasmonic layers thickness or overly strong light–plasmon interaction, most of the guided light is absorbed or scattered, leaving a weak transmitted signal. This reduces the measurable output and decreases the signal-to-noise ratio (SNR), making precise detection challenging. Additionally, high CL broadens the resonance peak, which diminishes the SR and its ability to detect small RI variations.

Thus, using Equation (11), the WS of 2000, 12,500, 11,333.33, 12,250, and 28,000 nm/RIU is obtained for the Basal, Jurkat, Hela, PC 12, and MDA MB 231 cells, respectively, corresponding to x-pol. Similarly, the WS of 1500, 11,000, 11,666.66, 12,000, and 25,500 nm/RIU is obtained for the Basal, Jurkat, Hela, PC 12, and MDA MB 231 cells, respectively, corresponding to y-pol. Thus, the average WS of 13,216.66 nm/RIU and 10,353.33 nm/RIU is obtained, corresponding to x-pol. and y-pol., respectively, which is relatively less when compared with the WS of the proposed biosensor at optimum thickness.

**Figure 10:**
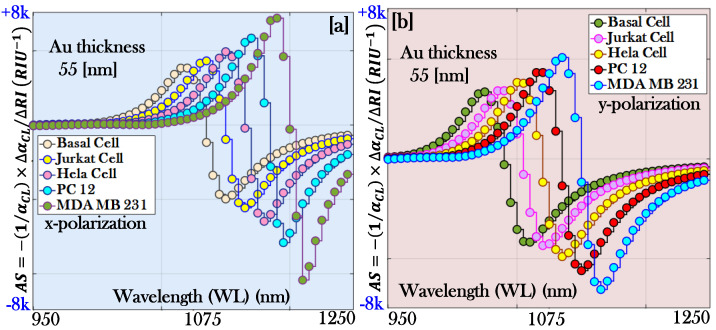
Amplitude sensitivity of the proposed biosensor with increased thickness of the plasmonic material Au (55 nm): (**a**) x-polarization and (**b**) y-polarization.

Now using Equation (12), the AS of 4334.67, 5124.22, 6423.91, 7224.22, and 7645.33 ${RIU}^{-1}$ is obtained, corresponding to cancerous cells Basal, Jurkat, Hela, PC 12, and MDA MB 231, respectively, as presented in Figure 10a for x-pol. Similarly, the AS of 3896.38, 4213.98, 5432.11, 6389.54, and 7162.52 ${RIU}^{-1}$ is obtained, corresponding to cancerous cells Basal, Jurkat, Hela, PC 12, and MDA MB 231, respectively, as presented in Figure 10b for y-pol. The peak value of the AS is obtained for the cancerous cells MDA MB 231 for both pol. modes.

Thus, it can be observed that on increasing the thickness of the plasmonic layer beyond its optimum value significantly affects the performance of the biosensor. Specifically, the CL increases, while the WS and AS decrease. This occurs because a thicker plasmonic layer reduces the penetration of the guided light into the sensing region, weakening the interaction between the core mode and the surface plasmons. Therefore, accurate computation and identification of the optimum plasmonic layer thickness are critical for achieving high performance in PCF–SPR sensors.

### 5.2. The Effect of Varying the Geometrical Dimension of the Air Holes for the Increased Thickness of the Plasmonic Material

In this section, the impact of varying the air hole dimensions (d) in the PCF by ±10% is investigated for analyzing the performance of the biosensor. Specifically, we investigate how such dimensional changes influence key sensor parameters, including CL and AS, for different biofluids such as the RI of MCF-7 and MDA-MB-231 cancer cells. Understanding these effects is important because the geometry of the air holes strongly affects the mode confinement, evanescent field distribution, and coupling with the plasmonic layer, all of which directly determine the sensor’s sensitivity and overall operational efficiency.

**Figure 11:**
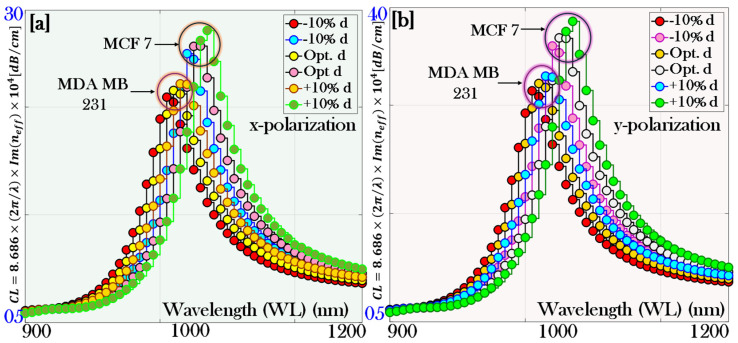
Comparison of confinement loss at optimized thickness and ±10% variation in dimension (d) of the air holes: (**a**) x-polarization and (**b**) y-polarization.

Figure 11a illustrates the variation of CL for the cancer cells MCF-7 and MDA-MB-231 under x-pol. For MCF-7, the optimized CL is 28.81 dB/cm, which increases to 29.54 dB/cm and decreases to 27.62 dB/cm when the air hole diameter (d) is increased and decreased by ±10%, respectively. Similarly, for MDA-MB-231, the optimized CL is 27.76 dB/cm, rising to 28.56 dB/cm and falling to 26.54 dB/cm for +10% and −10% variations in the air hole dimension, respectively.

Figure 11b illustrates the variation of CL for the cancer cells MCF-7 and MDA-MB-231 under y-pol. For MCF-7, the optimized CL is 38.29 dB/cm, which increases to 39.57 dB/cm and decreases to 37.18 dB/cm when the air hole diameter (d) is increased and decreased by ±10%, respectively. Similarly, for MDA-MB-231, the optimized CL is 34.81 dB/cm, rising to 35.63 dB/cm and falling to 33.43 dB/cm for +10% and −10% variations in the air hole dimension, respectively.

**Figure 12:**
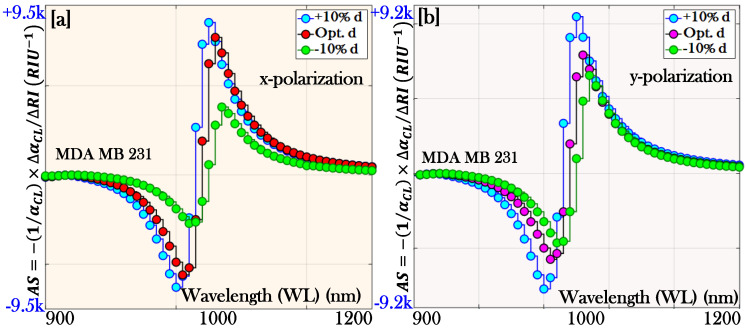
Comparison of amplitude sensitivity at optimized thickness and ±10% variation in dimension (d) of the air holes: (**a**) x-polarization and (**b**) y-polarization.

Similarly, the variation in the AS for the cancer cell MDA MB 231 can be obtained from Figure 12a,b. In Figure 12a the optimum AS for MDA MB 231 was 9158.29 RIU^−1^, which increased to 9238.45 RIU^−1^ and decreased to 9032.63 RIU^−1^ by changing the air hole dimension d by +10% d and −10% d, respectively, for x-pol. Similarly, in Figure 12b the optimum AS for MDA MB 231 was 9004.54 RIU^−1^, which increased to 9198.83 RIU^−1^ and decreased to 8928.51 RIU^−1^ by changing the air hole dimension d by +10% d and −10% d, respectively, for y-pol.

Thus, it can be observed that by varying the thickness of the plasmonic material beyond its optimum value significantly influences the sensing performance of the biosensor. A similar effect is also observed when the geometrical dimensions of the structure, such as the air hole diameter, are altered. In both cases, the biosensor parameters, including CL, WS, and AS, deviate from their optimal values, which can degrade overall performance. These observations clearly indicate that the optimized design parameters represent the most favorable operating conditions, under which the sensor achieves maximum efficiency, reliability, and sensitivity. Therefore, precise optimization of both plasmonic thickness and structural geometry is essential to ensure the stable and effective operation of PCF–SPR biosensors.

## 6. Discussion

The numerical analysis of the proposed quasi-D-shaped PCF–SPR biosensor demonstrates that combining Au and PMMA coatings within the sensing region significantly enhances plasmonic resonance characteristics, leading to high detection sensitivity for various cancerous biomarkers. The exhaustive investigation of structural and material parameters of the biosensor reveals that the plasmonic layer thickness critically influences the evanescent field penetration; increasing the Au layer beyond its optimum value raises CL and weakens light–analyte interaction, thereby reducing WS and AS. Similarly, geometric variations in air-hole dimensions affect mode behavior, highlighting the necessity of precise structural control during the real-time fabrication. The optimized configuration demonstrates superior performance compared to conventional SPR sensors. The quasi-D geometry facilitates efficient cancer fluid access and strong field coupling, making the design particularly suitable for biomedical sensing applications. Overall, the findings validate the proposed hybrid Au–PMMA PCF structure as a highly promising platform for label-free, real-time cancer cell detection. Table 8 presents a comparative analysis of the sensor parameters of previously presented sensor models with the proposed sensor model.

As it can be observed, the proposed hybrid Au–PMMA-coated sensor model exhibits markedly improved sensing performance compared with the Au-coated sensor parameters reported in refs. [64,65,66,67,68]. The proposed PCF–SPR sensor, incorporating a hybrid plasmonic layer composed of Au and PMMA, demonstrates superior performance relative to a conventional Au-coated sensor model. The sensing performance is comparatively evaluated against the Au configuration in terms of RW shift, CL, WS, AS, and SR. The results clearly indicate that the hybrid plasmonic configuration significantly outperforms the Au-based sensor. The hybrid structure enables stronger plasmonic field confinement at the metal–dielectric interface, larger RW shifts for identical RI variations, and enhanced overall sensitivity. In contrast, the Au-only configuration exhibits weaker LMI and limited resonance tunability, resulting in comparatively inferior sensing performance.

Finally, despite the promising performance of the proposed biosensor it has certain limitations that warrant further investigation. First, the present study is entirely numerical, and experimental validation is still required to confirm that the simulated sensitivity and resolution under practical fabrication and measurement conditions are not affected beyond a certain limit. Secondly, the real time fabrication of the proposed PCF with nanoscale Au and PMMA coatings poses technological challenges, including achieving uniform deposition and maintaining structural integrity during polishing. The sensor’s performance may also vary with temperature fluctuations, mechanical stress, and analyte infiltration dynamics, which were not considered in the current simulations. Finally, the biosensor is optimized for cancer cell fluid samples only; extending its functionality toward multi-analyte or label-free detection would enhance its clinical applicability. These aspects will be addressed in future experimental studies. Future research can focus on experimental realization of the design using advanced fabrication and coating techniques to validate its simulated performance. The integration of multi-analyte detection and artificial intelligence (AI)-assisted signal processing could further enhance its diagnostic accuracy and real-time detection capability. The hybrid plasmonic–polymer configuration also provides a versatile foundation for detecting other biomarkers and distinct analytes, extending its application beyond cancer diagnostics to broader biomedical and environmental sensing domains.

Finaly it can be concluded that the proposed PCF–SPR biosensor operates on the principle of RI modulation and therefore does not intrinsically exhibit biochemical specificity toward individual cancer cell types. The sensor response is governed solely by variations in the effective RI of the surrounding analyte, enabling label-free discrimination only when the RI contrast between different cancer cell fluids is sufficiently distinct. Accordingly, the cancer cell examples discussed in this work are presented to demonstrate the applicability of the proposed sensor within biologically relevant RI ranges rather than to claim cell-type-specific recognition. It is worth noting that enhanced specificity toward cancer biomarkers can be achieved through appropriate surface functionalization of the Au layer with selective bioreceptors, which will be analyzed as the future prospect of this research work.

## 7. Conclusions

The proposed quasi-D-shaped PCF–SPR biosensor, employing an Au plasmonic coating with a PMMA overlayer, demonstrates enhanced sensitivity to RI variations associated with cancer cells, indicating its suitability for detection applications. The systematic study of plasmonic layer thickness and air-hole dimension variations revealed that optimized structural parameters are critical for achieving maximum sensitivity and stability. Increasing the plasmonic thickness beyond the optimum leads to higher CL but reduced evanescent field penetration, while deviations in air-hole dimensions also degrade performance. Under optimized geometrical conditions, the sensor achieves a high WS of 31,000 nm/RIU and 29,500 nm/RIU for MDA-MB-231 cells in x- and y-pol., respectively, along with ultra-low resolutions of 3.22 × 10^−6^ RIU and 3.38 × 10^−6^ RIU. Furthermore, the biosensor maintains high AS values exceeding 9000 RIU^−1^. These findings confirm that the quasi-D-shaped PCF–SPR biosensor offers a robust, highly sensitive, and reproducible platform suitable for biomedical diagnostics, particularly in early cancer detection.

## Figures and Tables

**Table 1:** Refractive indices of cancer cell lines and their biophysical interpretation.

Cancer Type	Typical Cell (*n_cell_*)	Biological Reason for Higher/Lower RI	Ref.
Basal cell	1.38	moderately dense cytoplasm	[32]
Jurkat cell	1.39	suspended round cells, dense nuclei	[33]
HeLa cell	1.392	increased nucleic acid and protein content	[34]
PC 12 cell	1.395	neuroendocrine cells, rich in proteins	[35]
MDA MB 231 cell	1.399	aggressive line, high cytoplasmic density	[36]
MCF 7 cell	1.401	epithelial-like, dense organelles	[37]

**Table 2:** Mesh component classification of the proposed biosensor.

Elements	SiO_2_	Air Hole	Au	PMMA	Fluid Channel	Zeonex
Maximum element size (μm)	1.15	0.36	0.4645	0.4289	0.00018	0.007115
Minimum element size (μm)	0.0054	0.00135	0.0054	0.0054	3.6 × 10^−4^	0.00213
Growth rate	1.3	1.2	1.3	1.3	1.1	1.3
Curvature factor	0.3	0.25	0.325	0.3	0.2	0.3
Resolution	1.0	1.0	1.0	1.0	1.0	1.0

**Table 3:** Refractive indices of the cancer cells and their detailed explanation.

Cancer Type and Associated Cancer Cells	Refractive Index	Details of the Individual Cancer	Ref.
Skin cancer (Basal cell)	1.38	BCC is the most common type of skin cancer, originating in the basal cells of the epidermis. These cells have relatively lower RI that reflects basal cancer cells, which often have lower density and less optical scattering compared to more aggressive tumor cells. Detecting such small RI differences allows biosensors to differentiate between healthy and cancerous skin tissue [58,59,60].	[13]
Blood and bone marrow (Jurkat cell–T-cell leukemia)	1.39	Jurkat cells are derived from a patient with acute T-cell leukemia (T-ALL). They are suspension cells (circulating in blood). They have slightly higher RI than skin cancer cells that reflect their altered biochemical composition (proteins, nucleic acids, and lipids) [61].	[13]
Cervical cancer (Hela cells)	1.392	HeLa cells are one of the most famous immortalized cancer cell lines, derived from cervical carcinoma. Their RI is higher than that of Jurkat or basal cells, indicating denser cellular content and greater light scattering due to uncontrolled proliferation and altered membrane composition [62].	[13]
Adrenal gland cancer (PC-12)	1.395	PC-12 cells originate from a pheochromocytoma (a tumor of the adrenal glands medulla). These cells are neuron-like and are widely used as a model for neural and endocrine cancers. Their RI is intermediate between HeLa and breast cancer cells, reflecting unique cytoplasmic density and organelle composition [63].	[13]
Breast cancer (MDA-MB-231)	1.399	These are triple-negative breast cancer (TNBC) cells, one of the most aggressive subtypes of breast cancer. Their RI is significantly higher, correlating with dense packing of proteins, lipids, and nucleic acids inside the cells [64].	[13]
Breast cancer (MCF 7)	1.401	MCF-7 cells are a luminal A subtype of breast cancer, less aggressive than MDA-MB-231 but still malignant. They can be attributed to their larger size, higher cytoplasmic density, and lipid-rich content [65].	[13]

**Table 4:** Sensor parameters corresponding to various cancer cells for a 45 nm thick Au layer.

Cancer Cells and Respective RI	x-polarization		y-polarization		Shift	
	CL (dB/cm)	RW (nm)	CL (dB/cm)	RW (nm)	$\left|∆CL\right|$	$\left|RW\right|$
Basal (1.38)	10.12	954	13.12	951	3.0	3.0
Jurkat (1.39)	13.26	975	16.84	969	3.58	6.0
Hela (1.392)	19.84	1004	23.46	994	3.62	10.0
PC 12 (1.395)	23.42	1045	29.56	1032	6.14	13.0
MDA MB 231 (1.399)	27.76	1100	34.81	1083	7.05	17.0
MCF-7 (1.401)	28.81	1162	38.29	1142	9.48	20.0

**Table 5:** Polynomial constants for x-polarization.

Polynomial Constants	Value	Lower	Upper
$p_{1}$	6.4194 × 10^5^	3.9357 × 10^5^	8.9031 × 10^5^
$p_{2}$	−1.7757 × 10^6^	−2.4663 × 10^6^	−1.0850 × 10^6^
$p_{3}$	1.2288 × 10^6^	7.4866 × 10^5^	1.7090 × 10^6^

**Table 6:** Polynomial constants for y-polarization.

Polynomial Constants	Value	Lower	Upper
$p_{1}$	6.0856 × 10^5^	3.7457 × 10^5^	8.4255 × 10^5^
$p_{2}$	−1.6836 × 10^6^	−2.3343 × 10^6^	−1.0329 × 10^6^
$p_{3}$	1.1654 × 10^6^	7.1305 × 10^5^	1.6178 × 10^6^

**Table 7:** Sensor parameters corresponding to various cancer cells for 55 nm thick Au layer.

Cancer Cells and Respective RI	x-polarization		y-polarization		Shift	
	CL (dB/cm)	RW (nm)	CL (dB/cm)	RW (nm)	$\left|∆CL\right|$	$\left|RW\right|$
Basal (1.38)	15.23	968	16.27	962	1.04	6
Jurkat (1.39)	19.53	988	21.84	977	2.31	11
Hela (1.392)	27.84	1013	29.76	999	1.92	14
PC 12 (1.395)	32.42	1047	38.82	1034	6.40	13
MDA MB 231 (1.399)	36.81	1096	44.51	1082	7.70	14
MCF-7 (1.401)	43.54	1152	58.02	1133	14.48	19

**Table 8:** Comparison of sensing parameters with previously reported biosensors.

Polarization/ Year/ Design/Ref.	Fiber Type/ Coating	Structural Design	Simulation/ Experimental	Cancer Cell Type	WS (nm/RIU)	AS (RIU^−1^)	SR (RIU)
PCF/2021/[68]	One Dimensional (1D)/ SiO_2_ and TiO_2_	Sample layers sandwiched between two PCs	Simulation	MCF 7 Breast Cancer	74.5	NA	NA
				Hb in blood plasma	73.0	NA	NA
x-pol. and y-pol./EMD/ 2018/[69]	SiO_2_	Circular	Simulation	Hela Cell (x-pol.)	7916	NA	NA
				Hela Cell (y-pol.)	10,625	NA	NA
y-pol./Slotted EMD/ 2021/[27]	Au and SiO_2_	Bended	Simulation	MCF 7 Breast Cancer	7142.86	NA	1.4 × 10^−5^
				MCF 7 Breast Cancer	10,000	NA	
x-pol. and y-pol./EMD/ 2023/[26]	Au, MXenes, SiO_2_	Circular	Simulation and ML	MCF 7 Breast Cancer	10,714	NA	9.3 × 10^−6^
				MCF 7 Breast Cancer	13,071	NA	7.6 × 10^−6^
y-pol./D shaped/2023/[28]	Au	D shaped	Experimental	HER2 detection, Breast Cancer	28,100	NA	NA
y-pol./EMD/2025/ [29]	Au and TiO_2_	Two C-grooves shaped	Simulation	Blood, breast, and adrenal cancer	29,000	NA	3.45 × 10^−6^
y-pol./EMD/ 2024/[70]	Au, TiO_2_, SiO_2_	Circular shape with oblong air holes	Simulation	MCF 7 Breast Cancer	11,429	1251.18	8.75 × 10^−6^
				MDA-MB231			
x-pol./ Quasi D shaped/ 2025/ Proposed	Au, SiO_2_, PMMA, Zeonex	Quasi D shaped	Simulation	Basal	2100	5057.97	4.76 × 10^−5^
				Jurkat	14,500	6193.26	6.89 × 10^−6^
				Hela	13,666.66	7490.46	7.31 × 10^−6^
				PC 12	13,750	8202.36	7.27 × 10^−6^
				MDA MB 231	31,000	9158.29	3.22 × 10^−6^
y-pol./ Quasi D shaped/ 2025/ Proposed	Au, SiO_2_, PMMA, Zeonex	Quasi D shaped	Simulation	Basal	1800	5017.97	5.55 × 10^−6^
				Jurkat	12,500	6083.76	8.0 × 10^−6^
				Hela	12,666.66	6990.49	7.89 × 10^−6^
				PC 12	12,750	7862.54	7.84 × 10^−6^
				MDA MB 231	29,500	9004.54	3.38 × 10^−6^

## Data Availability

The original contributions presented in this study are included in the article/Supplementary Materials. Further inquiries can be directed to the corresponding author.

## Supplementary Materials

The following supporting information can be downloaded at: https://www.mdpi.com/article/10.3390/mi17010068/s1, Video S1: illustrate the electromagnetic field distributions of modes corresponding to x- and y-pol.
