# Supplementary figures and images for: Theoretical Investigation of Early Cancer Biomarker Sensing Using a PMMA–Gold Hybrid Quasi-D-Shaped Photonic-Crystal-Fiber-Based Surface Plasmon Resonance Biosensor
Source: Micromachines (Basel). 2025 Dec 31;17(1):68. doi: 10.3390/mi17010068 (PMC12844213; doi:10.3390/mi17010068)

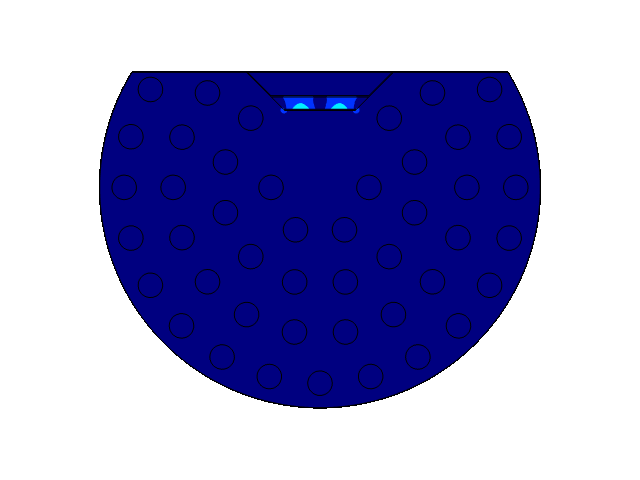

Supplement: Supplementary file 1 [file micromachines-17-00068-s001.zip › micromachines-4011819-supplementary.gif]
